# Messing Up the Cancer Stem Cell Chemoresistance Mechanisms Supported by Tumor Microenvironment

**DOI:** 10.3389/fonc.2021.702642

**Published:** 2021-07-20

**Authors:** Miriam Gaggianesi, Simone Di Franco, Vincenzo Davide Pantina, Gaetana Porcelli, Caterina D'Accardo, Francesco Verona, Veronica Veschi, Lorenzo Colarossi, Naida Faldetta, Giuseppe Pistone, Maria Rita Bongiorno, Matilde Todaro, Giorgio Stassi

**Affiliations:** ^1^ Department of Surgical Oncological and Stomatological Sciences (DICHIRONS), University of Palermo, Palermo, Italy; ^2^ Department of Health Promotion Sciences, Internal Medicine and Medical Specialties (PROMISE), University of Palermo, Palermo, Italy; ^3^ Pathology Unit, Mediterranean Institute of Oncology, Catania, Italy; ^4^ Department of Surgery, Villa Sofia-Cervello Hospital, Palermo, Italy

**Keywords:** cancer stem cells, tumor microenvironment, anticancer drugs, chemoresistance, targeted therapy

## Abstract

Despite the recent advances in cancer patient management and in the development of targeted therapies, systemic chemotherapy is currently used as a first-line treatment for many cancer types. After an initial partial response, patients become refractory to standard therapy fostering rapid tumor progression. Compelling evidence highlights that the resistance to chemotherapeutic regimens is a peculiarity of a subpopulation of cancer cells within tumor mass, known as cancer stem cells (CSCs). This cellular compartment is endowed with tumor-initiating and metastasis formation capabilities. CSC chemoresistance is sustained by a plethora of grow factors and cytokines released by neighboring tumor microenvironment (TME), which is mainly composed by adipocytes, cancer-associated fibroblasts (CAFs), immune and endothelial cells. TME strengthens CSC refractoriness to standard and targeted therapies by enhancing survival signaling pathways, DNA repair machinery, expression of drug efflux transporters and anti-apoptotic proteins. In the last years many efforts have been made to understand CSC-TME crosstalk and develop therapeutic strategy halting this interplay. Here, we report the combinatorial approaches, which perturb the interaction network between CSCs and the different component of TME.

## Introduction

Despite huge progress has been made in the development and optimization of anti-tumor therapies, cancer remains the second leading cause of death worldwide. Intra- and inter-tumor heterogeneity represents the main hurdle for cancer treatment. For this reason, the comprehension of the molecular and phenotypic differences among different cancer types may help to improve the prognosis of cancer patients upon therapy. Two models have been proposed to explain the origin of tumor heterogeneity (1). According to the stochastic model, each cell within the tumor mass can become tumorigenic by acquiring specific (epi)genetic alterations. Conversely, in the hierarchical model tumor heterogeneity arises from a subpopulation of cancer cells, termed cancer stem cells (CSCs), able to self-renew and differentiate into phenotypically and functionally distinct cells. CSCs share most of the features with normal stem cells, but their self-renewal capacity is typically deregulated (2, 3). Therefore, CSCs represent the roots which feed tumor initiation and sustain metastatic spread, therapeutic resistance and recurrence (4, 5). Chemotherapy is a pivotal treatment for solid tumors and aims to counteract all the active proliferative cells, including both healthy and malignant cells (6). Compelling evidence have demonstrated that CSCs are endowed with *i)* high expression of ATP-binding cassette (ABC) transporter and anti-apoptotic molecules, *ii)* aberrant activation of proliferative and survival signaling pathway and *iii)* a proficient DNA repair machinery are the main mechanisms inducing multidrug resistance (MDR) (1). Interestingly, recent studies have shown that tumor microenvironment (TME) could generate a protective niche for tumor cells from drugs, leading to chemoresistance. In addition to the intrinsic characteristics of CSCs, the interaction with the TME must be taken into account because it is involved in the regulation of signaling pathway and resistance to therapy, representing a potential target for novel therapeutic approaches (7). In this review, we will illustrate TME protective effects against chemotherapic drugs and the most updated strategies for targeted therapies alone or in combination to disrupt the CSCs/TME interaction.

## Cancer Stem Cells as a Shield to Elude Chemotherapeutic Agents

Different hypotheses have been made about the origin of CSCs, as a direct consequence of (epi)genetic alterations in the healthy stem cell compartment, or from progenitor/differentiated cells through the dysregulation of stemness-related pathways (8).

The pioneering studies conducted by Tilland McCulloch in early ‘60s demonstrated the existence of hematopoietic stem cells, opening the era of stem cell research (9). Later in 1994, Lapidot et al., provided the first evidence of CSC presence in acute myeloid leukemia (AML). AML cells were fractioned according to the expression of cell surface markers CD38 and CD34 and the obtained different subpopulations were injected into immunocompromised mice. They noticed that only the CD34^+^/CD38^-^ subpopulation was able to engraft in mice reflecting many features of human AML (10, 11). The first demonstration of CSC existence in solid tumors was provided in breast cancer (BC) (12) and later in brain, colon, thyroid and other tumors (13, 14), pointing out that cancer cell transplantation into immunocompromised mice is the gold standard assay to identify and characterize CSCs (15). Compelling evidence point out that CSCs are responsible for the failure of the conventional therapies, due to aberrant activation of signaling pathways, high expression of efflux transporters/anti-apoptotic molecules, and enhanced DNA-damage repair machinery (4, 16, 17).

### Stemness-Related Pathways Involved in CSC Chemoresistance

Deregulation of developmental and proliferative pathways, such as Hedgehog (HH), Wnt/β-catenin and Hippo, sustains CSC growth and chemoresistance (18). The HH pathway has been shown to regulate the properties of CSCs in various neoplasms through the up-regulation of stemness-related genes (Nanog, Oct4, Sox2 and Bmi1) (19, 20). In colorectal cancer (CRC) HH-GLI pathway activation fostered CSC survival and sustained *in vivo* growth and metastatic ability (21). In BC, the CD44^+^/CD24^-^ subpopulation isolated from tumor xenografts displayed high expression levels of HH signaling molecules compared to more differentiated cell subsets (22). In glioma, the activation of Notch and HH pathway mediated the resistance to temozolomide treatment in CD133^+^ CSCs (23). Aberrant activation of the Wnt/β-catenin signaling pathway has been mainly linked to development of CRC (24, 25) and detected also in other tumor types, as hepatocellular and BC (26). Recently, it has been demonstrated that knockdown of Wnt1 decreases the expression of CD44, Aldehyde dehydrogenase 1 (ALDH1) and Sca-1 stemness genes, thus leading to the reduction of CSC subpopulation and tumor sphere formation in BC cells (27). Several studies linked Wnt/β-catenin signaling and chemoresistance (28). The overexpression of Frizzled1 (FZD1), a receptor of Wnt ligands, increased ABCB1 transporter and mediates MDR in neuroblastoma and BC (29, 30). Moreover, LGR5, a Wnt target gene, promoted resistance to 5-fluoruracil (5-FU) treatment in CSCs (31, 32). Recent studies have revealed a complex crosstalk between Wnt and Hippo-YAP/TAZ pathways. Hippo pathway *via* YAP/TAZ activation led to the induction of CSC properties in BC cells (33). In a very elegant study, Cheung et al demonstrated that the Hippo kinases LATS1/2 and MST1/2 maintain Lgr5^+^ CSCs phenotype and sustain the activation of Wnt/β-catenin signaling pathway in CRC (34).

### Alterations of Apoptotic Pathways and DNA Damage Repair Machinery in Chemoresistant CSCs

Alterations of apoptotic pathways and DNA damage repair machinery are among the principal mechanisms underlying CSC-mediated chemoresistance. Apoptosis regulates tissue development and homeostasis and is finely regulated by a network of signals that are crucial for cell fate. The ratio between apoptotic and anti-apoptotic protein levels defines the sensitivity of malignant cells to apoptotic stimuli and contributes to CSCs resistance to anticancer treatments (35). A weakened expression of death receptors (DRs) was observed in CSCs from different tumors compared to differentiated counterparts. In AML, the CD34^+^ CD38^-^ stem-like subpopulation display a lower expression of FAS and FAS ligand (FAS-L) than CD38^+^ differentiated cells, triggering chemoresistance (36, 37). FAS and FAS-L reduced expression was also observed in glioma stem cells (GSCs) and the use of a synthetic FAS-L, Apo010, in combination with temozolomide induced apoptosis in glioblastoma (GBM) stem-like cells (38, 39). Moreover, the use of recombinant soluble TRAIL (TNF related apoptosis inducing ligand), in combination with bortezomib reduced the colony formation capacity of GSCs and impaired tumor growth in a mouse model of GBM (40). Unfortunately, the short half-life of soluble TRAIL in plasma reduces its efficacy. An interesting approach to overcome this effect is the use of TRAIL-engineered mesenchymal stromal cells, which induce apoptosis and curtail the colony forming ability of lung and breast cancer stem-like cells (41, 42). However, CSCs usually exhibited TRAIL resistance, due to c-FLIP over-expression. In BC and GBM, c-FLIP up-regulation sustained resistance to TRAIL therapy and the use of siRNA specific for c-Flip lessened self-renewal and tumorigenic potential of breast CSCs (43–45). The inhibitor of apoptosis (IAP) proteins were found to be over-expressed in CD133^+^ GBM stem cells compared to the CD133^-^ compartment and their inhibition, by using small molecules, enhanced apoptosis in γ-irradiated cells (46, 47). In GBM patients, the IAP protein, survivin, was demonstrated to be mainly expressed in patient-derived GBM stem cells compared to differentiated cells, with a predominant localization in the core of tumor mass and associated with the expression of CD133, SOX2 and MELK (48). In addition, our group demonstrated that highly chemoresistant colorectal CSCs are characterized by the autocrine production of IL-4 that boosts survivin expression (49, 50). On the other hand, the dysregulation of Bcl2 family, composed by anti-apoptotic (Bcl2, Bcl-xL and Mcl-1) and pro-apoptotic (Bak, Bax, Bid, Bim, Bic, Noxa and PUMA) factors, has been found in CSCs (51). In particular, the stem-like compartment expressed higher expression level of Bcl2 and Bcl-xL compared to differentiated cancer cells (46, 52). Moreover, in breast CSCs the activation of αvβ3/Src/Slug signaling pathway leads to inactivation of PUMA through SLUG, a PUMA repressor. The pharmacological inhibition of Src with dasatinib enhanced PUMA expression levels, reducing self-renewal and colony formation capacity and increasing sensitivity to apoptosis (53, 54). On the contrary, the interaction of PUMA with Bcl2 and Bcl-xL limited its anti-apoptotic activity and a combined treatment of Src and Bcl2 inhibitors increased apoptosis, thus reducing chemoresistance (55).

Chemotherapeutic drugs mainly target differentiated tumor cells, while sparing CSCs, characterized by a highly efficient DNA damage response (DDR) system able to repair DNA damage induced by radio- and chemotherapies (56, 57). In accordance, cisplatin (CIS) treatment led to an enrichment of CSC subpopulation in ovarian and lung cancers, confirming that chemotherapy efficiently eliminates rapidly dividing differentiated/progenitor cells (58, 59). DNA damage promoted the activation of ataxia-telangiectasia-mutated (ATM), Rad17, Chk1 and Chk2 checkpoint proteins. Experimental evidence showed that CD133^+^ GSCs are radio-resistant compared to CD133^-^ tumor cells, due to a more efficient checkpoint protein activation in response of DNA damage (60). Another study reported that GBM stem cells after irradiation increase the expression levels of L1CAM (CD171), which in turn up-regulates NBS1, an important component of MRN complex implicating in the early activation of ATM in response to DNA damage (61). Knockdown of L1CAM reduced the activating phosphorylation of ATM and Chk2 in response to IR-induced DNA damage, sensitizing GBM stem cells to radiation and reducing *in vitro* tumor sphere formation (62). In addition to GSCs, alteration of DDR pathway has been described in CSCs from different tumor types, including CRC (63). CD133^+^ lung cancer cells are resistant to ionizing radiation treatment due to an up-regulation of genes involved in double strand break repair, such as Rad51, BRCA1 and Exo1 (64). Moreover, invasive CD133^+^ stem-like cells isolated from pancreatic cancer cell lines displayed higher expression levels of gene involved in the BRCA1-mediated DNA repair pathway and resistance to gemcitabine (GEM) treatment compared to CD133^-^ subpopulation (65). In a syngeneic p53^null^ mice mammary gland tumor model, the Lin^-^/CD29^High^/CD24^High^ subpopulation was characterized by increased expression levels of DDR and DNA repair genes (66). Furthermore, Liu et al. demonstrated that CSCs, isolated from BRCA1-mutant BC cell lines, displayed resistance to PARP inhibitors and were characterized by the overexpression of RAD51. The use of a shRNA targeting RAD51 sensitized triple negative BC cells to olaparib treatment (67).

Another mechanism of CSC resistance to anticancer therapies is represented by the up-regulation of detoxifying enzymes and drug efflux pump expression levels. ALDH superfamily is responsible for oxidizing aldehydes to carboxylic acids and retinol to retinoic acid allowing the detoxification from drug and the reactive oxygen species (ROS). ALDH1 is the main isoform of the ALDH superfamily enzymes and is one of the markers used for the identification of the CSCs (68–71). In BC patients, ALDH1-positive CSCs were selected after neoadjuvant treatment and their presence within the tumor could predict resistance to chemotherapy (72). In breast CSCs, the resistance to doxorubicin and paclitaxel treatment is related to the over-expression of ABCB1 efflux pump (73). Moreover, ABCB1 confers resistance to carfilzomib in multiple myeloma stem cells (74). Indeed, high expression levels of ABCB5 were found in malignant melanoma initiating cell resistant to doxorubicin treatment (75) (**Table 1**).

**Table 1:** Cancer stem cell biomarkers correlated to chemoresistance.

CSC markers	Stemness-related pathways	Tumors	References
CD133	Hedgehog (HH)	CRC	21
CD44^+^CD24^-/low^ Lin^-^		BC	22
CD133		Glioma	23
CD133	Notch	Glioma	23
TOP-GFP^high^ CD133^high^	Wnt/β-catenin	CRC	25
Lgr5			32
Lgr5			31
			24
		BC	26
			27
ALDH activity, SP			30
		Neuroblastoma	29
CD44^+^CD24^-/low^	YAP/TAZ	BC	33
Lgr5		CRC	34
	**Apoptotic molecules**		
CD34^+^/CD38^-^	FAS/FAS-L	AML	36
		Pancreatic	37
CD133		Glioma	38
			39
CD133	TRAIL	GBM	40
CD133			44
CD133		BC	43
ALDH activity			45
CD133	IAP	GBM	22
			47
CD133, SOX2	Survivin	GMB	48
		CRC	49
CD133	Bcl2 family	GBM	22
		Haematopoietic disorders	54
CD44^+^CD24^-/low^		BC	53, 55
	**DNA damage repair machinery**		
CD133	Chk1/2	Glioma/GBM	60
SOX2			62
CD44v6 TOP-GFP^hig^		CRC	63
	ATM	GBM	61
SOX2			62
CD133	Rad51	Lung	64
ALDH activity		BC	67
CD133	BRCA1	Lung	64
CD133		Pancreatic	65
	**Detoxifying enzymes /Drug efflux molecules**		
CD44^+^CD24^-/low^	ALDH1	BC	72
CD44^+^CD24^-/low^ and CD133	ABCB1	BC	73
ALDH activity		Myeloma	74
CD133	ABCB5	Melanoma	75

## The Cancer Stem Cells-Tumor Microenvironment Interaction: a Hidden Hurdle in Chemotherapy Efficacy

**Figure 1:**
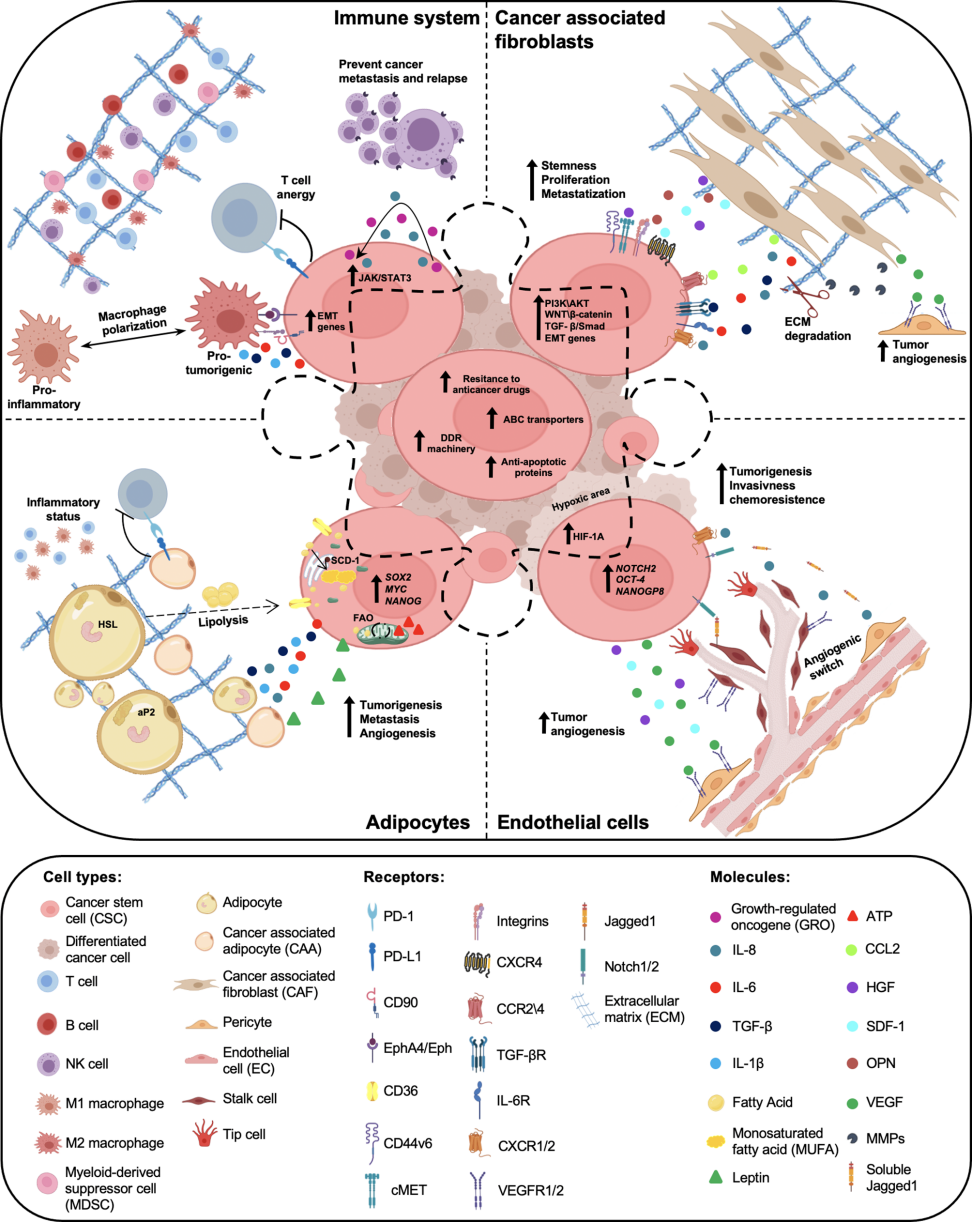
Crosstalk between cancer stem cells (CSCs) and tumor microenvironment (TME) components. Within the tumor mass, a subpopulation of cancer cells, called cancer stem cells (CSCs), are endowed with high resistance to anticancer therapies, due to elevated expression levels of ABC transporters, anti-apoptotic proteins and a proficient DNA damage repair (DDR) machinery. Tumor microenvironment (TME), mainly composed by cancer associated fibroblasts (CAFs), adipocytes, immune and endothelial cells, has a key role in the maintenance of CSC peculiarities. Cytokines and chemokines produced by both CSCs and TME cells boost cancer cell growth, prompt chemoresistance and promote tumor progression and relapse.

CSCs require the cooperation of surrounding microenvironmental cells to promote tumor initiation, metastasis formation and drug resistance. Recent evidence highlighted the importance of TME cell education and recruitment as essential events for tumor dissemination. In fact, cancer cells prime stromal cells, which in turn maintain and boost CSC subpopulation (76). In particular, CSC features are regulated by autocrine and paracrine interactions between tumor cells and TME, mainly composed by extracellular matrix, cancer-associated fibroblasts (CAFs), cancer-associated adipocytes (CAAs), immune and endothelial cells. In addition to the intrinsic characteristic of CSCs, the understanding of tumor-TME cell interactions could provide actionable candidates for the development of novel therapeutic approaches (**Figure 1**).

### CAF Role in Inducing CSC-Mediated Resistance to Therapy

CAFs are the major component of TME involved in the complex network of tumor-stroma evolution and tumorigenesis (77, 78). Several studies demonstrated that CAFs can originate from the activation of resident fibroblast or derive from the conversion of adipocytes, endothelial cells, pericytes and bone marrow-derived mesenchymal stem cells (79). CAFs provide numerous molecules, soluble factors and proteases playing an important role in ECM synthesis or remodeling, but also pro-inflammatory cytokines, chemokines, and growth factors. Notably, CAF secreted factors are involved in a tight crosstalk with CSCs, governing their self-renewal capacity, plasticity and chemoresistance (80–85). Several *in vitro* experiments highlighted that CAFs guarantee a CSC reservoir in different tumors, such as breast, lung, colorectal, gastric and liver, enhancing stem markers expression (CD44, Sox2, Bmi-1), sphere formation, self-renewal and sustaining CSC pool expansion (86–90). Moreover, CAF conditioned medium influenced the tumorigenic behavior and the aggressiveness of CSCs (91). For these reasons, CAFs represent a cellular subtype on which particular attention is being paid to predict patients’ outcome and to design new target therapies.

CAF-secreted factors, which include chemokines, cytokines, growth factors, proteins and exosomes, influence and sustain CSCs aggressiveness by modulating their stemness features. CCL2 supported CSC self-renewal activating NOTCH signaling pathway and the co-injection of CAF and breast CSCs into the mammary fat pads of NOD/SCID/IL-2Rg-null mice enhanced CSC tumorigenic potential, unveiling the CCL2 driving role in BC (83). In addition, SDF-1 interacting with its receptor (CXCR4), highly expressed on CSC surface, regulated stem phenotype through the activation of Wnt/β-catenin and PI3K/AKT signaling pathways and boosted the proliferation of CD44^+^/CD24^-^ BC cells (92). In agreement, CXCR4^+^ cells were more prone to reach the stem phenotype and properties, in comparison to CXCR4^-^ cells (90).

Among the variety of cytokines and growth factors secreted by CAFs, a great number of studies highlighted the IL-6 and IL-8 essential role in the maintenance of stem-like features of cancer cells and in the promotion of tumor growth, metastasis formation, and chemoresistance (93, 94).

CAFs also supported the aggressive behavior of cancer cells through the secretion of TGF-β. In different tumor types, TGF-β induced the acquisition of a stem-like phenotype, promoted EMT and chemoresistance *via* the activation of TGF-β/Smad signaling pathway (88, 95).

Hepatocyte growth factor (HGF), another important molecules secreted by CAFs, promotes cancer cell invasiveness. In hepatocellular carcinoma, HGF sustained cancer cell stemness through the activation of MET/FRA1/HEY1 cascade (89). Our group recently demonstrated that HGF, SDF-1, and OPN released by CAFs were able to reprogram CD44v6^-^ progenitor cells in metastatic CD44v6^+^ CSCs by activating Wnt/β-catenin and PI3K/AKT signaling pathways (90).

Synthesis and remodeling of extracellular matrix (ECM) represents an important function of CAFs in TME. Malanchi et al. demonstrated in a murine BC model that lung fibroblasts, activated by infiltrating CSCs, produce periostin which boosts Wnt pathway sustaining metastatic colonization (96). Moreover, in a syngeneic BC mouse model S100A4^+^ lung CAFs secreted VEGF-A and tenascin-C, which mediate angiogenesis and CSC survival, respectively (97). In addition to the production of ECM components, CAFs secreted metalloproteases, a family of enzymes able to degrade and remodel ECM, favoring cancer cell invasion (98).

Numerous studies highlighted that CAFs can support CSC chemoresistance in different solid tumors. Co-culture experiments performed with freshly isolated colorectal CSCs showed that CAFs secrete high levels of TGF-β2 and IL-6, which in turn prompt the transcription of GLI-2, promoting resistance to 5-fluorouracil/oxaliplatin (5-FU/oxa) treatment (99). In breast and lung tumors, the CD10^+^/GPR77^+^ CAF subpopulation secreted both IL-6 and IL-8, which induce CSC enrichment and chemoresistance to CIS treatment (100). Moreover, in triple negative BC mice models, cancer cells reprogrammed CAFs through the secretion of HH ligand. CAFs, in turn, triggered the acquisition of chemoresistance through FGF5 secretion and the production of fibrillar collagen (101). In head and neck small cellular cancer, CAF-secreted periostin bound PTK7, a receptor expressed on cancer cell surface, favoring CSC invasion and proliferation through the activation PTK7–Wnt/β-Catenin signaling pathway. Notably, PTK7/periostin interaction enhanced erlotinib chemoresistance and the formation of lung metastasis (102). Recently, our group demonstrated that in colorectal CSCs CAF-secreted cytokines confer resistance to PI3K/AKT inhibitors (103) (**Table 2**). Given the key role of CAFs in both CSC maintenance and drug refractoriness, the use of therapeutic strategies blocking CAFs-CSCs crosstalk could improve patients’ survival. In resistant BC cells, trastuzumab treatment activated an IL-6/STAT-3/NF-κB inflammatory loop, which correlates with the expansion of the CSC subpopulation. The administration of an anti-IL-6 receptor antibody reverted the stem-like phenotype of tumor cells (104). Zong et al. reported that the use of MEDI5117, an anti IL-6 antibody, in combination with chemotherapy or gefitinib impairs tumor growth in mice injected with NSCLC cells. MEDI5117 also displayed robust activity against trastuzumab-resistant HER2 tumor cells by targeting the CD44^+^/CD24^-^ population (119).

**Table 2:** Molecular mechanisms prompting cancer stem cell resistance to standard and targeted therapies.

	Mechanism of resistance	Tumor	Molecule secreted	Drug	References
**Cancer associated fibroblasts (CAFs)**	GLI-2 enhanced expression	CRC	TGFβ2 , IL-6	5-FU/oxa	99
	NF-kB pathway	BC/lung	IL-6, IL-8	CIS	100
	Wnt/β-catenin pathway	head/neck	periostin	erlotinib	102
	PI3K/AKT and MAPK pathway	CRC	HGF, SDF-1, OPN	PI3K/AKT inhibitors	90, 103
	STAT3/NF-kB pathway	BC	IL-6	trastuzumab	104
**Cancer associated adipocytes (CAAs)**	High MVP expression levels	BC	CM from adipocytes	doxorubicin/ 5-FU/ paclitaxel	105
	MAPK pathway	BC	IL-4	arimidex/ docetaxel+BKM120	106
	AKT/MAPK pathways	BC	Leptin	5-FU	107
	MAPK and AKT pathways	CRC	Leptin	5-FU	108
	AMPK/mTOR/JNK pathways	BC	Resistin	doxorubicin	109
	Up-regulation of ABCG2	BC	CXCL1	doxorubicin	110
**Endothelial cells (ECs)**	Notch pathway	Lymphoma	Jagged-1	doxorubicin	111
	Notch pathway	CRC	Jagged-1	5-FU/oxa	112
	High c-Met expression levels	GBM		bevacizumab	113
	HIF/VEGF pathways	CRC	VEGF	bevacizumab	114
	Increase of intratumoral hypoxia	LLC		sunitinib	115
	VEGF-independent angiogenesis	Pancreatic cancer	FGFs	DC101 (anti-VEGFR2)	116
	VEGF-independent angiogenesis	RCC	IL-8	sunitinib	92
	NF-kB pathway	RCC	IL-6	sunitinib	117
	AXL and Met signaling	RCC		sunitinib	118

A novel strategy to counteract IL-6 downstream pathway is represented by the use of specific oligonucleotide decoy specific for STAT3, which display encouraging anticancer effects. In EGFR inhibitors-resistant NSCLC cells, the treatment with a cyclic STAT3 decoy (CS3D) impaired *in vitro* proliferation and tumor formation (120). Likewise, AZD9150, a STAT3 antisense oligonucleotide, sustained antitumor activity in lymphoma and NSCLC preclinical models. Based on these promising results, AZD9150 was used as single agent in a Phase I clinical trial including patients with advanced lymphoma and NSCLC (121). Moreover, the double inhibition of IL-6 and IL-8 in combination with docetaxel in CD10^+^/GPR77^+^ CAFs impaired tumor growth in a patient-derived xenograft (PDX) model of BC (100). In human BC PDX, the use of a specific antibody against IL-8 receptor, CXCR-1, or an inhibitory molecule against to CXCR-1 and CXCR-2, repertaxin, favored the eradication of CSC pool, thus impeding tumor progression. In particular, ALDH^+^ and CD24^-^/CD44^+^ levels were reduced by ≥ 20% in 4/17 and 9/17 patients (122). Two independent research groups described that the use of CXCR-2 inhibitors, AZ13381758 and SB225002, suppresses tumor progression and hampers chemotherapy resistance in BC and pancreatic adenocarcinoma, respectively (123, 124).

The use of smoothened inhibitors (SMOi), in combination with docetaxel, in triple negative BC PDX and in a Phase I clinical trial (EDALINE) reduced metastasis formation and displayed clinical benefits, respectively (101). In addition, vismodegib, a HH inhibitor, triggered apoptosis and decreased both CAF and CSC proliferation in breast, colon and prostate cancer (125–127). In head and neck cancer, the combination of anti-PTK7 and erlotinib highly reduced tumor growth compared to single agent treatment (102).

In gastric cancer, TGFβ1 neutralizing antibody or TGFβR inhibitor (Ki26894) reduced the side population fraction, able to exclude fluorescence dye, even in presence of CAF conditioned medium (88). Alike, treatment with AMD3100 (plerixafor), a CXCR4 antagonist, blocked SDF1/CXCR4 interaction leading to a regression of CSC subpopulation in breast, colon and prostate cancer (125–127). A Phase I study on a cohort of cancer patients with worse prognosis showed that treatment with a cMET pan-inhibitor, capmatinib (INC280), displays anticancer activity in 8/44 patients (128). In preclinical studies, treatment with WNT/β-catenin inhibitors, iLGK974, Wnt-C59, and cyclosporin A, impaired CSC survival in different cancer types (129–131). In this context, we have recently demonstrated that the use of a variant of BMP7 with enhanced stability (BMP7v) induced the differentiation of CD44v6^+^ cells, suppressed Wnt pathway activity and sensitized CSCs to standard and target therapies (132). Recently, we demonstrated that cytokines secreted by CAFs boosted resistance to PI3K/AKT inhibitors in colorectal CSCs and this protective effect was overcome by the triple targeting of Her2, PI3K and MEK (103).

Interestingly, new therapeutic approaches focus on the direct depletion of CAFs. The targeting of FAP^+^ CAFs could represents a new promising target therapy (133). In agreement, FAP^+^ CAF inhibition and depletion with the use of the dipeptidyl peptidase inhibitor PT100 led to a reduction of the crosstalk between CAF and pro-tumorigenic immune or endothelial cells by enhancing oxa treatment efficacy in colon cancer mice models (134). Interestingly, FAP could be used as an antigen for CAR-T anticancer treatment strategy. For instance, treatment with CAR-T against FAP^+^ CAFs promoted growth arrest in *in vivo* models of lung cancer xenografts and syngeneic murine pancreatic cancers (135).

### Adipocyte-Released Factors Strengthening CSC Chemotherapy Refractoriness

Adipose tissue (AT) is a specialized soft connective tissue consisting of about 90% of adipocytes cells and for the remaining part by adipose derived stem cells (ADSCs), endothelial cells, pericytes, fibroblasts and immune cells (macrophages, dendritic cells, lymphocytes). AT can be divided according to anatomic localization in three different subtypes: subcutaneous, visceral and intramuscular. In addition, adipose depots may be sub-classified in white (WAT) and brown AT (BAT), which is characterized by a dark color due to the presence of vessels and a high number of mitochondria (136). For these reasons, BAT is mainly implicated in thermogenic regulation, maintaining the appropriate balance between energy storage and consumption. AT originates from the mesoderm, whose cells give rise to adipocyte and the myogenic lineages. Specifically, white adipocytes derived from the adipogenic MYF5 negative cells, whereas brown adipocytes from myogenic MYF5 positive cells (137). White and brown cells could be discriminated in accordance with the expression of specific markers, with white adipose cells expressing leptin and S100B, lacking UCP-1 expression, and brown adipose cells characterized by PPAR gamma and UCP-1 (138). In the last years, WAT, which was traditionally considered as an energy storage tissue, due to the triglycerides and cholesterol contained in intracellular droplets, has been demonstrated to represent the biggest human endocrine organ, with the production and release of hormones, growth factors, cytokines and adipokines. Accordingly, a conspicuous secretion of these factors is observed in obesity conditions (139, 140). Nowadays, obesity represents a global health problem and constantly increases in all countries of the world (141, 142). It has been demonstrated that overweight and obesity correlate with the onset of several solid tumors, including esophagus, pancreatic, colon, breast, endometrium, ovarian and kidney, suggesting an association between these conditions and tumor initiation (143, 144). In obese subjects, adipocytes increase their dimension (hypertrophy) both in subcutaneous and visceral ATs, whereas only visceral is characterized by an increase of adipocyte number (hyperplasia) (145).

In obese conditions, WAT secrete high amount of hormones, adipokines and pro-inflammatory cytokines, such as leptin, IGF-1, HGF, TNF-α, IL1β, IL-4, IL-6, IL-8, plasminogen activator inhibitor 1 (PAI-1) and CCL-2. This promotes both a chronic inflammatory state and a tumor-permissive microenvironment, which in turn induce tumorigenesis, neo-angiogenesis *via* VEGF release and metastatic progression (106, 134, 146–150). Furthermore, adipokines locally recruit monocytes, macrophages, lymphocytes and other immune cells, which increase the inflammatory status in the AT particularly in obese subjects (151). The adipocytes’ role in TME has been broadly studied in the context of BC. The established crosstalk between BC cells and the close AT cells increases the production of cytokines with proinflammatory activity. Picon-Ruiz et al. demonstrated that tumor cells, after exposure to proinflammatory cytokines, are characterized by the activation of ALDH1 and an increment of mammosphere formation capacity, which are correlated with the increase of CSC number and metastasis formation in *in vivo* settings. These processes are driven by Src oncogene, which activates the transcription of *SOX2*, *MYC* and *NANOG*, well-known stem cell markers (152). In agreement with these observations, we have previously demonstrated that the release of IL-4 sustains breast CSCs invasion, tumorigenic potential, and drug resistance (106). Moreover, in obese conditions, adipocytes released elevated levels of leptin, which trigger the activation of many stemness-related molecular pathways, as Notch, Wnt/B-catenin, OCT4,SOX2, Nanog and ALDH1 up-regulation (153–156). In intestinal epithelial cells, the activation of Wnt pathway determines the expansion of crypt stem cells and favors progenitor proliferation (153–156). Breast CSCs harness higher lipid metabolism than differentiated cancer cells and used long chain fatty acids as an energy source (157). This population is characterized by an increased β-oxidation activity, which produces numerous metabolic intermediates used in ATP production (154). Recent studies have shown that ovarian and colorectal CSCs retain a high amount of fatty acids within lipid droplets to maintain their stem-like features (158). This population is rich of monounsaturated fatty acids (MUFAs), generated by stearoyl-CoA desaturase-1 (SCD1), which are metabolic markers of CSCs (159, 160). The inhibition of SCD1 decreased ovarian CSC phenotype, impairing the expression of SOX2, Nanog and Oct4, sphere forming capacity and tumorigenic potential (161–163). In melanoma, lipids released by adipocytes induced metabolic reprogramming, enhancing cell proliferation (164).

In addition, tumor cells prime peritumoral adipocytes, boosting intense lipolysis. In fact, these adipocytes, called cancer associated adipocytes (CAAs), show both *in vitro* and *in vivo* smaller cell sizes and irregular shapes with an expanded ECM and over-expression of collagen IV. They are also characterized by an increased secretion of proinflammatory factors and numerous high-energy metabolites, free fatty acids, ketone bodies, pyruvate, and lactate (165, 166). Several studies show that CAAs activate Wnt/β-catenin pathway, leading to the loss of terminal adipocyte differentiation markers such as adiponectin (APN), resistin, hormone-sensitive lipase (HSL) and adipocyte protein 2 (aP2) (166). In particular, CAAs have some characteristics of the senescence-associated secretory phenotype (SASP), such as the release of proinflammatory factors (167). CAA-released leptin determined the activation of STAT3-CPT1-fatty acid β-oxidation (FAO) in CSCs, with an increased use of fatty acids as an energy source. The *in vivo* blocking of this signaling pathway led to a reduction of stem-like features and a re-sensitization of breast tumor cells to chemotherapy (107).

Recent studies showed that obesity could be associated with treatment-related toxicity (168), thus, lower doses of chemotherapeutic drugs are administered to obese patients, compromising therapy efficacy and leading to resistance development (169, 170). These observations highlighted that the body max index (BMI) is not the appropriate parameter to determine the dose of chemotherapy, because it does not take into account the altered pharmacokinetics and pharmacodynamics in obese patients. A meta-analysis revealed that obese patients treated with full chemotherapy doses, estimated using actual body weight, showed lesser toxicity compared to normal weight subject (171). Lehuédé et al. observed that adipocytes promote *in vitro* resistance to doxorubicin, paclitaxel and 5-FU in BC cells and this phenomenon is amplified by adipocytes isolated from obese women (105). It has been demonstrated that the adipocytes, to accomplish their protective effect on BC cells treated with doxorubicin, increase the production and secretion of resistin mediating AMPK/mTOR and JNK signaling pathway activation (109). In addition, doxorubicin may influence adipocyte functions, deregulating adipokine secretion and thus altering lipogenesis and lipolysis (170). Yeh et al. observed that pre-adipocytes promote doxorubicin resistance in triple negative BC by secreting CXCL1, which determines over-expression of ABCG2 (110). Moreover, high concentrations of leptin increased colorectal CSCs survival and the resistance to 5-FU treatment (108) (**Table 2**).

In addition to cytotoxic drugs, adipocytes are implicated in the resistance to multiple therapies, including radiotherapy, hormonal therapy, immunotherapy, and chemotherapy (172–174). CAAs expressed high levels of PD-L1 and in turn protected cancer cell from the anti-tumor activity of CD8^+^ T lymphocytes (151). Of note, the use of immune checkpoint inhibitors in BC displays limited efficacy, probably due to the presence of surrounding AT. The inhibition of adipogenic processes increased anti PD-L1 or anti PD-1 activity (175). Moreover, it was demonstrated that IL-6 secreted by mammary adipose tissue up-regulated Chk1 signaling pathway in BC cells, promoting resistance to radiotherapy (138). Therefore, the targeting of tumor-released factors which induce the activation of adipocytes in CAAs could improve patient outcomes. In BC cachectic patients, the secretion of miR-155 by tumor cells restored adipocyte metabolism, reducing PPARγ expression levels, and was associated with tumor progression. The administration of propranolol impaired the release of exosomes containing miR-155, thus restoring PPARγ in adipocytes (176, 177).

Moreover, targeting the metabolic dependence of cancer cells on adipocytes could be a therapeutic strategy to lessen tumor progression (178). In melanoma cells, the treatment with fatty acid transport protein 1 inhibitor impaired the invasive capacity of tumor cells promoted by adipocyte conditioned medium (179). In addition, CD36 inhibition in ovarian cancer cells reduced their *in vitro* and *in vivo* invasive capabilities sustained by CAAs (180). Moreover, Masko et al. pointed out that the combination of standard treatments with drugs interfering with adipocyte metabolism, like statins, has promising therapeutic relevance in prostate cancer treatment (181, 182). The administration of a high fat diet, instead of a normal one, in mice treated with diethylnitrosamine promoted hepatocellular carcinoma development, increasing STAT3 activation and IL-6 production. This phenotype was counteracted using acyclic retinoids (183, 184). Metformin is an anti-hyperglycemic agent, indicated for obesity-related type 2 diabetes, which determines the inhibition of the hepatic gluconeogenesis pathway, through the activation of AMPK. Moreover, metformin reduced the circulating levels of androgen, estrogen, insulin and sensitized BC cells to chemo and radiotherapy through a selective killing of stemness compartment (NCT02874430) (185–187).

### Immune Cells Rewiring Therapies

Immune system is an interesting network composed of specialized immune cells (ICs), cytokines, chemokines, and lymphoid organs, which, all together, contribute to immune response. The principal function of immune system is to discriminate “self” from “non-self” components. In TME, ICs affect both cancer development and immunological surveillance, influencing patients’ clinical outcome (188). ICs are classified in effector and non-effector cells, with the first category including natural killer (NK) cells, B and T lymphocytes, involved in the adoptive immune response along with the killing of cancer cells. The presence of T cytotoxic CD8^+^, T helper 1 (Th1) CD4^+^, B and NK cells within the TME is associated with a positive patients’ outcome in many cancers (189, 190). It is well known that NK cells act directly on the tumor cells, hampering their proliferation and dissemination. Compelling evidence demonstrated that NK cells eradicate CSCs at mestatatic sites, preventing tumor progression and relapse (191–194). Differently, the reduction of NK cells was associated with a worse outcome (195).

Non effector cells include antigen-presenting cells (APCs), regulatory T cells (Treg), tumor-associated macrophages (TAMs) and myeloid-derived suppressor cells (MDSCs), which support tumor growth, progression, and dissemination, hampering immune response.

The most abundant IC subset within the TME is represented by TAMs, which modulate the innate immune response in the context of tumor. TAMs own a phenotypic plasticity, thus transit from M1 to M2 phenotypes, and *viceversa*. M1 TAMs are involved in activated proinflammatory pathway and counteract tumor growth, while the M2 are engaged in anti-inflammatory response, largely promoting angiogenesis and tissue remodeling and sustaining tumor progression (196, 197).

Several studies highlighted that M2 TAMs are characterized by the expression of specific markers, such as CD163 and CD206 (198). In BC the release of TNF-α, IL-6 and IL-1β in the TME sustained M2 macrophages, which boost tumor initiation, dissemination and metastasis formation (199). In addition, Rodriguez-Garcia et al. highlighted the role of folate receptor β (FRβ) in TAMs cells. They demonstrated that immunosuppressive M2 TAMs expressing FRβ promote tumor progression in a mouse model of ovarian cancer, pointing out FRβ as a potential therapeutic target in combination with chemo- or immunotherapy (200). Furthermore, the expression of FRβ in M2 TAMs correlated with a poor prognosis also in pancreatic cancer (201). A recent study showed that BC cells, through TNF-α and IL-1β releasing in TME, induce the production of CCL8 by pro-tumorigenic TAMs and this crosstalk correlates with worse outcomes (202). These data confirm that M2 phenotype of TAMs plays a pivotal role in sustaining tumor growth and therefore could be a potential target. Overall, many studies highlighted that TAMs are responsible, in addition with other factors and cells available in the TME, for the increase of CSC subpopulation, leading to chemotherapy resistance. The induction of EMT and the over-expression of stem cell markers, such as CD90/Thy1 and EphA4, mediated the crosstalk between CSCs and TAMs. In addition, in different types of cancers and in particular in BC, the maintenance of a stem-like phenotype is also correlated to the presence of M2 macrophages in the TME (203, 204). In osteosarcoma, Xue-jing Shao et al. described the contribution of CD209^+^ M2 macrophages in tumor initiation and CSC maintenance, corroborating the possibility that the blockage of M2 macrophages depletes CSC subpopulation in the tumor bulk and, at the same time, inhibits tumor progression (205).

In liver cancer, the activation of oncoprotein Yes-associated protein (YAP) in CSCs correlated with both tumorigenesis and TAM recruitment, indicating that the blocking of M2 macrophage or YAP could be an efficacious therapeutic strategy (206).

In BC the inflammatory process predisposes to the malignant transformation, inducing the release chemokines, such as IL-8 and growth-regulated oncogene (GRO), which activate JAK/STAT3 pathway and in turn maintain CSC-like cell phenotype (207). Larionova et al. dissected the contributions of M2 macrophages in chemoresistance, showing that the depletion of M2 macrophages or M2-to-M1 re-polarization improves therapy efficacy of conventional cytotoxic drugs and/or immunotherapy, enhancing immune response (197).

It is widely demonstrated that cytotoxic chemotherapeutic drugs weaken immune system homeostasis (208). Simultaneously, over the last ten years, several studies highlighted the effects of chemotherapeutic agents regarding increased immunogenicity of human cancer cells and the role of chemotherapies in activating antitumor immune responses (209, 210). Different studies shed light on the role of NK cells within tumors and the influence of TME and chemotherapy on innate lymphoid cells. The high presence of NK cells in the TME correlate with an increased patients’ survival in different types of cancer, such as HER2-positive and triple negative BCs (211). It has been demonstrated that NK function could be regulated by chemotherapy (212). Recent studies have reported that different chemotherapeutic compounds, such as GEM, positively regulate NK cell functions. In lung cancers, the use of low-dose GEM enhanced the release of INF-γ and at the same time activated NK cells (213). In *in vivo* models of pancreatic cancer, the use of GEM as adjuvant chemotherapy improved mice overall survival with a reduction of tumor burden bulk. Thereafter, GEM induced a decrease of MDSCs and, on the other hand, increased the anti-tumor capability of NK cells (214, 215). In the last decades, to eradicate cancer cells many therapeutic strategies were focused on the re-activation of ICs, in particular of T cells. Cancer immunotherapies comprehend different approaches including the immune checkpoint blockade, with anti programmed death 1 (PD-1)/PD-ligand 1 (PD-L1)/cytotoxic T lymphocyte antigen 4 (CTLA4) antibodies, and adoptive cellular therapies (216). The following data illustrate that the use of cytotoxic chemotherapy combined with immunotherapy could block signaling factors or targets essential for CSC-mediated tumor progression and dissemination (190, 217).

In order to counteract fast cancer cell proliferation and enhance immune response, Orecchioni et al. tested the synergic effect of 5-FU, cyclophosphamide (CPX) or vinorelbine in combination with checkpoint inhibitors. In immunocompetent mice, the treatment with all the three chemotherapic drugs influenced the number of circulating ICs, in particular reducing MDSCs, APC cells, Treg, whereas increasing NK cells. The combination of chemotherapy and anti PD-L1 in mice injected with triple negative BC and B cell lymphoma cells reduced tumor growth and metastasis formation compared to the control group (218).

Many studies have been carried out to characterize the immunomodulatory properties of GEM. In pancreatic cancer, GEM induced a decrease in MDSCs and Treg, albeit did not counteract effector lymphocytes. Although GEM influences infiltrating ICs, generating an unfavorable condition for tumor growth, it was not sufficient as single agent and needed to be combined with immunotherapy to enhance immune response (219). In fact, in *in vivo* models GEM in combination with immunotherapy reduced the number of immunosuppressive cells, enhancing CD8^+^ T cells and promoting tumor cell elimination (220). These results pointed out that GEM is an immune checkpoint inhibitor-compatible drug, and this combination treatment reactivates the immune response with the goal of killing active proliferating cells (221).

Chemotherapy resistance is nowadays a sensitive issue that led several scientists to look for the causes of this phenomenon and the possibility to counteract the failure of chemotherapy drugs linked with the CSC subgroup. In NSCLC adenocarcinoma, the use of pemetrexed firstly stimulated the host antitumor immunity and simultaneously induced *in vitro* immunogenic cancer cell death (ICD), leading to improved antitumor immune response (222). In the KEYNOTE–021G trial, the use of pemetrexed and carboplatin (CARB) plus anti PD-1 antibody promoted immune response through the recruitment of infiltrating T cells, the reduction of APC cells, as well as elicited ICD in patients affected by NSCLC, improving their clinical outcome (NCT 02039674).

It has been demonstrated that CIS, oxa and CARB are able to stimulate antitumor immunity by promoting the enhancement of CD8^+^ T and APC cells with concomitant down-regulation of Treg and MDSC subpopulations. This effect, prompted by platinum derivatives drugs, improved the sensitivity of tumor cells to immunotherapy (223).

In bladder cancer cell lines, the use of CIS increased the expression level of PD-L1 through the activation of c-Jun, one of the activator protein-1 (AP-1) subunits, *via* ERK1/2. These data showed that chemotherapy in combination with immunotherapy (anti PD-L1) preempts cancer relapse blocking AP-1 oncogene factor (224).

The Keynote-407 multicentric study investigated the use of immune checkpoint inhibitors alone or in combination with CARB and paclitaxel in squamous NSCLC patients. The response rate and median progression-free survival increased in patients treated with checkpoint inhibitors plus chemotherapy instead of placebo groups (NCT02775435). The use of these combinatorial regimes recruited T, NK, and APC cells with a concomitant reduction of MDSCs and Treg in the TME (225).

The results of a Phase III clinical trial, which includes recurrent inoperable or metastatic triple-negative BC patients, reported that pembrolizumab plus chemotherapy (paclitaxel, GEM) improved the median progression-free survival (NCT02220894) (226). Moreover, in an ongoing Phase III randomized trial in patients affected by metastatic CRC the use of chemotherapy (FOLFOX) in combination with immunotherapy (atezolizumab, anti PD-L1) was tested in order to hamper cancer progression and improve immune system response, in particular cytotoxic CD8^+^ T cells (NCT02912559) (227). All these data highlighted that immunotherapy, in association with standard chemotherapy, has erupted as a novel therapeutic strategy to counteract tumor growth and chemoresistance.

In addition to the use of immune checkpoint inhibitors, another promising therapeutic approach is the chimeric antigen receptor T-cell (CAR-T) therapy. This methodology is based on the use of patients T cells engineered with vectors carrying a CAR specifically expressed on cancer cells. This genetic modification allows T cells, after re-infusion in patients, to efficiently recognize and kill cancer cells (228). To date, five different generations of CARs, characterized by differences in their intracellular domain, have been developed. Specifically, the first generation of CARs presented only the CD3ζ domain, which in the second generation was conjugated with a costimulatory domain, such as CD28 or 4-1BB, to improve their proliferation and cytotoxic potential. The third and fourth generation differed from the second generation for the addition of CD137/CD134 or IL-2 inducer domain, respectively. To improve CAR-T proliferation and survival, the last generation display a STAT3 inducer domain in combination with CD3ζ-CD28 and IL-2 inducer (229, 230). Promising data showed that CAR-T cells, engineered for the most abundant surface antigen expressed on CSCs, efficiently target cancer cells mainly in liquid tumors. CAR-T cell-based clinical trials displayed huge remission rates in patients with B cell hematologic malignancies (231). In the ELIANA trial, children and young patients with refractory B-cell acute lymphoblastic leukemia (ALL) were infused with autologous T cells engineered with a CD19 CAR (CTL019, tisagenlecleucel), achieving durable remission with transient toxic effects (NCT02435849) (232). The anti CD19 CAR-T cell therapy also displayed remarkable results in adult patients with diffuse large B-cell lymphoma (DLBCL) (NCT02445248) (233). Despite the encouraging results obtained in the treatment of hematological tumors, limited successes have been reached with solid ones. This is probably due to the immunosuppressive role of TME and the heterogeneous expression of targetable antigens (234). Nevertheless, several CAR-T clinical trials have been approved for the treatment of solid cancers. The high expression levels of EpCAM have been associated with local growth and dissemination in different cancers, including breast and colorectal tumors (230). Zhang et al. described that use of CAR-T cells, targeting EpCAM^+^ cancer cells, induces tumor strinkage in *in vivo* CRC models (235). Moreover, in a Phase I clinical trial the use of a CD133 CAR-T cells induced, after the first infusion, the reduction of tumor growth and the partial remission or stable disease for the treatment in hepatocellular, pancreatic and CRC patients (NCT02541370) (236). Besides hitting cancer cells, CAR-T could also be engineered to target components of TME. In murine ovarian carcinoma cell lines, the use of CAR-T targeting FRβ induced a selective depletion of M2 TAMs and, at the same time, led to the recruitment of inflammatory cytokine and precursor myeloid cells. Despite the clinical benefits obtained in term of durable remission, the majority of CAR-T cell therapies displayed high grade toxic effects, such as cytokine-release syndrome and neurotoxicity (237, 238). Therefore, the next milestone on CAR-T cell therapies is the optimization of clinical approaches and engineering strategies to improve safety and efficacy.

### Role of Tumor Angiogenesis in Chemotherapy Failure

The aberrant and rapid growth of cancer cells requires a continuous demand of nutrient and oxygen, which generate hypoxic area in the TME. To restore an adequate oxygen supply, CSCs boosted HIF-1A expression levels which mediate the secretion of VEGF-A, SDF-1 and HGF, recruiting VEGF receptors (VEGFRs)-expressing endothelial cells (ECs) and promoting tumor angiogenesis (239–241). Through this process, VEGF signaling activates the proliferation and survival of ECs, determining the increase of vessel permeability and supporting the metabolic needs of cancer cells (242). Moreover, the VEGF secreted by CSCs recruited mesenchymal stem cells inducing their differentiation into ECs (243).

These observations indicate that CSCs play a fundamental role in determining the TME through an important crosstalk with mesenchymal cells and ECs associated with the tumor. In normal conditions, angiogenesis, which has key role during embryonic development and tissue repair, is finely regulated by a poise between pro- and anti-angiogenic factors (244). This process is characterized by a dynamic and complex sequence of events which involve two main cells type: proliferating stalk cells and the highly invasive and motile endothelial tip cells. At the end of vessel formation, pericytes and vascular smooth muscle cells are recruited to stabilize newly formed blood vessels (245). The alteration of normal angiogenesis is a hallmark of cancer which leads to important changes and transformation inside TME and is connected with tumor progression (246). In 1971, Folkman used for the first time the expression “tumor angiogenesis” to describe blood vessel sprouting mediated by activated ECs nearly tumor mass (247). The new tumoral vessels are characterized by chaotic organization and weak interactions between pericytes and ECs favoring vascular leakiness, which is one of the most important barriers for efficient drug delivery in solid tumors (245, 248, 249). Several studies described that CSCs could trans-differentiate and promote the formation of new vessels without the recruitment of ECs. In the 1999, Maniotis et al. described for the first time this phenomenon, called vascular mimicry, in melanoma (250). Thereafter, other groups described the trans-differentiation of CSCs in ECs and pericytes in other tumors, like GBM, colon, and BC (4, 243, 251–253). Calabrese et al. described a close ECs-brain CSCs interaction in the perivascular niche, which maintains the self-renewal capacity of CD133^+^ stem-like cells and supports xenograft tumor growth. The treatment with anti-angiogenic drugs impaired CSC features (254). In a 3D system, brain ECs secreted IL-8 which promotes the expression of stem cell markers and boosts the invasive potential of patient-derived GBM cells (255). Moreover, ECs isolated from different organs increased the stem-like phenotype of CRC cells and the expression levels of OCT4 and NANOGP8 mediated by AKT activation (256). Many studies highlighted the key role of NOTCH signaling pathway in ECs/CSCs cross-talk, which prompts stem-like phenotype and tumor progression in cancer cells (257).

In GBM, the juxtacrine signaling between NOTCH ligand-expressing ECs and tumor cells exposing Notch1 receptor boosted *in vitro* and *in vivo* growth of cancer stem-like cells (258). Cao et al. pointed out that FGF4, produced by lymphoma cells, induces the expression of Jagged-1 on ECs, which in turn promotes Notch2 activation in cancer cells, increasing their tumorigenic and invasive capacity (111). A similar mechanism has been described in breast CSCs (259, 260). In a complementary manner, ECs released a soluble Jagged-1 which activates Notch signaling in colorectal CSCs, enhancing their tumorigenic and metastatic potential (112).

The below reported studies pointed out that ECs not only promote stem-like phenotype in cancer cells, but also play a key role in the resistance to chemotherapy. The activation of Notch pathway triggered by ECs confers resistance to doxorubicin treatment in aggressive lymphoma cells (111). Moreover, EC conditioned medium drove refractoriness to 5-FU and oxa treatment in colorectal CSCs (112). Therefore, the possibility to counteract chemoresistance mediated by ECs/CSCs interaction could ameliorate the management of cancer patients. Compelling evidence showed that anti-angiogenic treatments not only contrast the formation of new blood vessels, but also improve the quality of existing blood vessels, enhancing blood perfusion and consequently the exposure of cancer cells to chemotherapeutic treatments (261). The majority of anti-angiogenic therapies is represented by monoclonal antibodies directed against EC membrane molecules, while other compounds targeted intracellular components, inhibiting their activation (261).

In the last decades, numerous anti-angiogenic drugs have been tested in clinical trials and approved by the FDA. Bevacizumab is a humanized monoclonal antibody directed against VEGF-A, approved in 2004, and used in clinic for treatment of different tumors, such as GBM, colorectal, ovarian and BC (262, 263). Bevacizumab prevents the VEGF-A/VEGFR interaction and thus impairs the activation of VEGF signaling pathways in ECs. *In vivo* studies have shown that bevacizumab inhibited the spouting of blood vessel, induced the regression of newly formed vessels, and normalized the morphology of preexisting ones to improve the administration of cytotoxic chemotherapy (242). However, the treatment with bevacizumab did not display significant improvement of patients’ overall survival in advanced BC (264). The inefficacy of bevacizumab treatment was also observed in colorectal and brain tumors due to the increased expression levels or activation of alternative angiogenic factors and signaling pathways, respectively (113, 114). In particular, Lu et al. demonstrated that in GBM CSCs, VEGF inhibits cell invasiveness by blocking HGF receptor (Met)/VEGFR2 interaction and recruiting PTP1B phosphatase, which promotes Met dephosphorylation. The treatment with bevacizumab led to Met signaling pathway activation and to the acquisition of a mesenchymal-like phenotype in GBM CSCs (265). Although angiogenesis inhibition initially reduce tumor growth and prevent metastasis formation, these effects are transitory and associated with tumor relapse and recurrence (266).

There are several explanations for the failure of anti-angiogenic therapies. One possible cause is the induction of intra-tumoral hypoxia related to decreased number of blood vessels and the over-expression of HIF-1A, which promotes and sustains CSC features and paradoxically reactivate neo-angiogenesis (267, 268).

These observations suggest that anti-angiogenic therapy used as single agent not only favors tumor growth and progression, but also induces therapy resistance. In fact, angiogenic inhibitors induced deep changes in vascular morphology involving the down-regulation of junction proteins and a reduction of pericyte number and functionality (115).

Another important limiting factor of anti-angiogenic drug efficacy is the activation of VEGF-independent pro-angiogenic signaling pathways in pancreatic tumors (116).

Other possible strategies able to interfere with tumor angiogenesis consist in the use of tyrosine receptor kinase inhibitors molecules (269). Sorafenib is an inhibitor of numerous tyrosine kinases, including Ras and VEGFR family and platelet-derived growth factor receptor β (PDGFR-β). In a Phase III study, the treatment with sorafenib increased the overall median survival of hepatocellular carcinoma patients (270). Moreover, in the DECISION trial patients with radioactive iodine-refractory thyroid cancer treated with sorafenib display an improved progression free survival compared to the placebo group (NCT00984282) (271). Unfortunately, mechanisms of resistance to sorafenib treatment similar to those described above for bevacizumab have been reported (272, 273). In addition, the intratumoral hypoxia generated by sorafenib treatment enhanced the expression of PD-L1 in cancer cells and the recruitment of TAMs (274).

Another tyrosine kinase inhibitor used as anti-angiogenic molecule is sunitinib, which targets PDGFR, VEGFRs and c-kit. Suninitib has been approved for the treatment of imatinib-resistant gastrointestinal stromal tumor (GIST) and metastatic renal cell carcinoma (RCC), displaying an increased response rate compared to placebo patient group (275). Nevertheless, patients rapidly acquire resistance to treatment (276). Huang et al. generated a sunitinib-resistant RCC xenograft model and observed high microvessel density together with increased serum levels of IL-8, suggesting that patients with elevated IL-8 levels display intrinsic resistance to sunitinib (277). Moreover, sunitinib treatment induced a stem-like phenotype and refractoriness in RCC cells through the activation of PAK1/NF-*κ*B/IL-6 signaling axis (117). In addition, the chronic administration of sunitinib in RCC cells promoted EMT, invasion and angiogenesis *via* the activation of MET and AXL. This sunitinib-induced phenotype was suppressed by cabozantinib treatment (118) (**Table 2**).

Based on the poor clinical efficacy of VEGF pathway inhibitors, in the last years alternative strategies have been tested to impair tumor angiogenesis. Small molecules (rebastinib) and monoclonal antibodies (MEDI3617, demcizumab, enoticumab and MEDI0639) targeting ANGPT2/TIE2 and Notch ligand–receptor interactions have been tested and approved for the treatment of advanced solid tumors (278, 279).

Given that CSCs can activate ECs through different stimuli, the simultaneous targeting or the subsequent multiple targeting of several angiogenic factors could represent an important perspective in the innovation of anti-angiogenic therapies avoiding the above described resistance mechanisms (261). In particular, in many clinical trials the treatment with anti-angiogenic compounds displays clinical effects only in early stage, due to a ‘selection’ of functional vessels among the newly tumor vessels. Therefore, this treatment-induced ‘therapeutic window’ could be an advantage for the administration of standard and targeted therapies (278).

Another important therapeutic strategy used to counteract the resistance to the combination of anti-angiogenic and cytotoxic drugs is the metronomic chemotherapy, based on the continuous administration of low chemotherapy doses. This approach hinders CSC/TME interactions, targeting both cancer cells and tumor-associated ECs (261, 280).

## Concluding Remarks and Future Perspectives

Despite the great advances made in early diagnosis and the development of targeted therapies, which increase patients’ survival rates, the metastatic disease remains incurable. This is mainly due to primary or acquired resistance to chemotherapeutic drugs and the presence of TME. Compelling evidence highlights that the inefficacy of anti-cancer therapy results from the refractoriness of a subpopulation of tumor cells, called CSCs, which are endowed with stem-like features including tumor-initiating and metastasis formation capabilities. In addition to the intrinsic characteristics of CSCs, interactions with TME are crucially involved in the resistance to chemo and targeted therapies. The mechanisms sustaining CSC/TME crosstalk and the limitations of targeting this complex signaling network have been comprehensively described in this review. Specifically, the reasons of treatment failure using the most recently available compounds targeting both CSCs or TME components have been reported. Of note, CSC plasticity and ability to adapt to the metabolic demand are the major hurdles in targeting CSC/TME interplay. Therefore, additional studies are needed to develop potential promising strategies to overcome cancer progression and drug refractory.

## Author Contributions

MG, SDF, and GS conceived and wrote the manuscript. VDP, GPo, CDA, FV, VV, LC, NF, GPi, MRB, and MT wrote the manuscript.All authors contributed to the article and approved the submitted version.

## Funding

This work was supported by grants from AIRC (21492) to MT and AIRC IG (21445) and PRIN (2017WNKSLR) to GS.

## Conflict of Interest

The authors declare that the research was conducted in the absence of any commercial or financial relationships that could be construed as a potential conflict of interest.
